# Selective pressure modulation of synaptic voltage‐dependent calcium channels—involvement in HPNS mechanism

**DOI:** 10.1111/jcmm.12877

**Published:** 2016-06-08

**Authors:** Ben Aviner, Gideon Gradwohl, Alice Bliznyuk, Yoram Grossman

**Affiliations:** ^1^ Department of Physiology and NeurobiologyFaculty of Health SciencesBen‐Gurion University of the NegevBeer ShevaIsrael; ^2^ Department of PhysicsJerusalem College of TechnologyJerusalemIsrael

**Keywords:** hyperbaric pressure, voltage‐dependent calcium channel, high‐pressure neurological syndrome

## Abstract

Exposure to hyperbaric pressure (HP) exceeding 100 msw (1.1 MPa) is known to cause a constellation of motor and cognitive impairments named high‐pressure neurological syndrome (HPNS), considered to be the result of synaptic transmission alteration. Long periods of repetitive HP exposure could be an occupational risk for professional deep‐sea divers. Previous studies have indicated the modulation of presynaptic Ca^2+^ currents based on synaptic activity modified by HP. We have recently demonstrated that currents in genetically identified cellular voltage‐dependent Ca^2+^ channels (VDCCs), Ca_V_1.2 and Ca_V_3.2 are selectively affected by HP. This work further elucidates the HPNS mechanism by examining HP effect on Ca^2+^ currents in neuronal VDCCs, Ca_V_2.2 and Ca_V_2.1, which are prevalent in presynaptic terminals, expressed in *Xenopus* oocytes. HP augmented the Ca_V_2.2 current amplitude, much less so in a channel variation containing an additional modulatory subunit, and had almost no effect on the Ca_V_2.1 currents. HP differentially affected the channels' kinetics. It is, therefore, suggested that HPNS signs and symptoms arise, at least in part, from pressure modulation of various VDCCs.

## Introduction

Humans, as most terrestrial mammals, are sensitive to hyperbaric pressure (HP). Pressure is a thermodynamic variable affecting the kinetics and steady‐state equilibrium of biological processes. Membrane phospholipids fluidity, ion channels, receptors, enzymes and other proteins functions are all potential targets for HP effects [for review, see 1]. Exposure of humans to HP (usually above 1.0 MPa) causes a constellation of signs and symptoms known as the high‐pressure neurological syndrome (HPNS). HPNS is the major problem associated with HP environment, as it occurs due to the effects of pressure *per se* 2, 3. Divers at depth above 90 msw may exhibit various symptoms, such as dizziness, nausea, tremors, vision and auditory disturbances, decrements in locomotion 4, 5 and cognitive performance 3, 6, 7, 8, 9, changes in electroencephalography (EEG) and sleep disorders 10, myoclonus 5, convulsions and a loss of consciousness (for review, see 11). Alteration in synaptic transmission is a plausible explanation for the HPNS (for review, see 12). Indeed, HP suppressed synaptic activity in most preparations. This suppression may occur *via* modulation of postsynaptic ionotropic receptors activity 13, 14, decreased AP amplitude 15, slowed kinetics 16, 17, depression of neurotransmitter release 18, 19, 20, 21 and modulation of its quantal release mechanism 22, 23, 24 and decreased vesicle fusion 13, 19. Most of these synaptic processes are known to be Ca^2+^ dependent. Earlier studies on crustacean neuromuscular synapses that examined the relationship between [Ca^2+^]_o_, excitatory post synaptic potential (EPSC) amplitude and facilitation 25, 26, 27 have suggested that pressure depresses Ca^2+^ influx rather than intracellular removal of Ca^2+^. Further support to this notion was the observations that low [Ca^2+^]_o_ partially mimics the effects of HP 20, 27 and high [Ca^2+^]_o_ can antagonize to some extent HP depression of current amplitude 15, 25, 28. In fact, modulation of presynaptic Ca^2+^ currents at HP has been already suggested 15, 29, 30. We, therefore, postulated that the major mechanism by which HP alters synaptic transmission is the modulation of Ca^2+^ influx into the presynaptic terminals through voltage‐dependent Ca^2+^ channels (VDCCs).

Various VDCC subfamilies are known, characterized by their electrophysiological and pharmacological traits: Ca_V_1.1‐4 (L‐Types), Ca_V_2.1 (PQ‐type), Ca_V_2.2 (N‐type), Ca_V_2.3 (R‐type) and Ca_V_3.1‐3 (T‐types), comprising the α_1_, α_2_δ, β and γ subunits 31, 32. The major difference between the channels results from the variation in the α_1_ subunit, which holds the ion conducting pore, the voltage sensor, the channel gating section and the known sites of channel regulation by second messengers, drugs and toxins 32. The α_2_δ, β and γ subunits have a modulatory effect on the ionic flux *via* α_1_ (for review, 33, 34), including its kinetic properties and voltage dependence. For example, the β_2a_ subunit slows channel inactivation in many subunit combinations. On the other hand, the coexpression of α_2_δ subunits 35, 36 and γ subunits 37 has a smaller functional effect. Lately, it has been suggested that the γ_2_ subunit is regulating the Ca_V_2.2 indirectly by counteracting Gβγ‐mediated effects such as slowing of activation and voltage‐dependent inactivation 38. Notwithstanding, a functional recombinant channel does not always require expression of all subunits.

Early findings of HP effects on VDCC currents were indirectly obtained (for review, see 39) from various preparations 27, 40, 41, 42, 43, 44. The sensitivity of the Ca_V_2.2 channel to HP 40, 41 was suggested, while the Ca_V_2.1 channel was rendered HP resistant 13, 17. Furthermore, Talpalar *et al*. 28 have postulated, based on mathematical modelling of experimental synaptic depression at HP, that rat dentate gyrus synapse is composed of pressure‐sensitive (probably Ca_V_2.2‐dependent) and pressure‐resistant (probably Ca_V_2.1‐dependent) independent modules of releasable vesicles pools.

In another attempt to study the HP selectivity of real currents, we have lately recorded extracellularly two components of Ca^2+^ currents in frog presynaptic terminals 15. Partial pharmacologic identification has suggested that a fast component is N‐type like and a slow component is probably one of the L‐type channels. Hyperbaric pressure differentially affected the currents; the fast Ca^2+^ currents being highly depressed, while the slow Ca^2+^ currents were much less inhibited.

The difficulty in positively identifying the Ca^2+^ currents in *ex vivo* experimental tissues, the presence of more than one type of current in each neuron either in the presynaptic terminals or soma and dendrites, the diversity of channels in various preparations and the technical difficulties in performing the pressure experiments have presented us with a major challenge. We have, therefore, embarked on a long‐term study that was aimed at overcoming these obstacles: direct measurement of VDCC currents by expressing the genetically identified cRNAs of the channels in frog oocytes under HP conditions. Recently, we have performed such a study for the first time on VDCCs currents of Ca_V_1.2 and Ca_V_3.2 30, demonstrating selective and sometimes transient HP effects on the channels: Ca_V_1.2 being potentiated, while the Ca_V_3.2 is depressed.

In the present report, we extended our study to include two additional VDCCs, Ca_V_2.1 and Ca_V_2.2, which are mainly, but not exclusively, present at the neuronal presynaptic terminals. It is hoped that comprehensive understanding of the behaviour of each VDCC at HP will enable us to refine a model of activity 39 based on known channels spatial distribution along the neurons. This could elucidate the HPNS mechanism and may enable us to reduce or even eliminate its short‐ and long‐term consequences.

## Materials and methods

### Oocytes extraction and cRNA injection

Oocytes of a *Xenopus laevis* mature female frog were surgically extracted from its ovary and treated with 1.5 mg/ml collagenase for 30–60 min. to remove connecting tissue. Suitable oocytes were sorted out by size, quality and developmental stage (VI), and kept in NDE96 solution containing (in mM): 96 NaCl, 2 KCl, 1 MgCl_2_, 1 CaCl_2_, 2.5 sodium pyruvate; 50 μg/ml gentamycin; 5 HEPES pH 7.5. Handling of frogs and oocytes extraction procedure were approved by the Ben‐Gurion University of the Negev's ethics committee for the care and the use of animals and are in compliance with international laws and policies.

cRNAs of the subunits of PQ or N‐type Ca^2+^ channels (Ca_V_2.1 or Ca_V_2.2, respectively) were synthesized from human, rat, mouse and rabbit cDNA by *in vitro* transcription with T7 or SP6 Amplicap High‐Yield Message Maker Kit (Epicentre Technologies, Madison, WI, USA). Oocytes were then injected with the specific cRNA mix (2.5 ng) encoding for the pertinent subunits to express Ca_V_2.1 or Ca_V_2.2 and were kept in an incubator for 4–5 days at 18°C in NDE96 solution. The following subunits were used: α_1A_ +β_3_+α_2_δ, comprising the Ca_V_2.1; and α_1Β_+β_3_+α_2_δ or α_1Β_+β_3_+α_2_δ+γ_2_, comprising the Ca_V_2.2.

### Electrophysiological recordings

**Figure 1:**
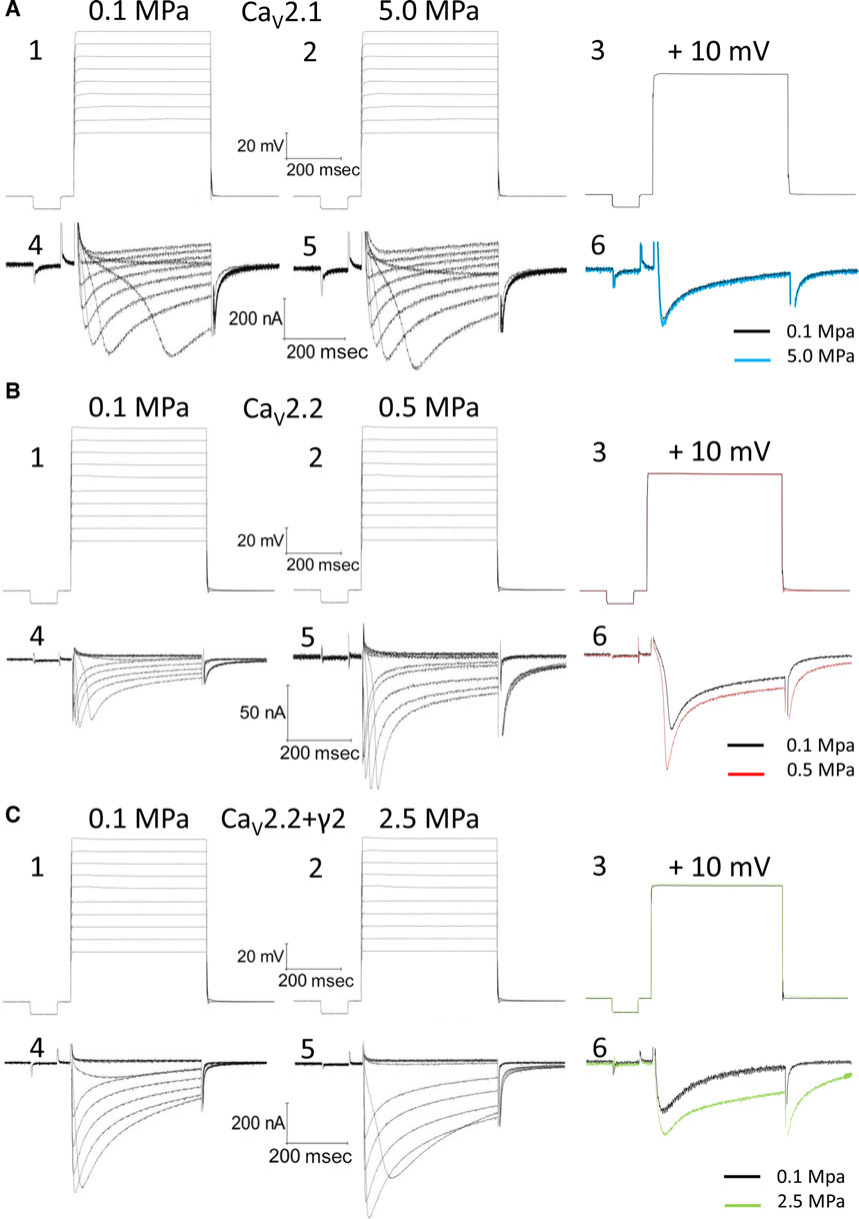
Ba^2+^ currents recorded in Ca_V_2.1 (**A**), Ca_V_2.2 (**B**) and Ca_V_2.2_+γ2_ (**C**) channels. A1‐3, B1‐3, C1‐3, depolarization steps for A4‐6, B4‐6 and C4‐6, respectively. A4‐5, B4‐5, C4‐5, currents at 0.1 and at HP, the exact pressure is indicated. A6, B6, C6, superimposed single current traces recorded under normobaric and hyperbaric conditions, generated by identical superimposed depolarization to 10 mV.

Four to five days after injection, the oocytes were placed in a specially designed bath, and two‐electrode voltage clamp experiments with 10‐mV increments and 5‐sec. interval between −70 and 40 mV were performed inside a compression chamber, utilizing an AXOCLAMP 2B amplifier (Molecular Devices, Axon Instruments, Inc., CA, USA), WinWCP pulse generating software by Strathclyde University, Axon Instruments DIGIDATA 1322A, and AxoScope 9.2 software. Sharp glass electrodes were fabricated using Sutter Instrument P‐1000 micropipette puller, filled with 3 M KCl, tip resistance <1.5 MΩ. The oocytes were penetrated by the electrodes, and only then the bath was carefully inserted into the chamber, slid onto an electric socket with preinstalled wires crossing the chamber wall. While in the chamber, each oocyte was continuously perfused with a Ba^2+^ solution containing (in mM): 20–40 Ba(OH)_2_, 50 NaOH, 2 KOH and 5 HEPES, titrated to pH 7.5 with methanesulfonic acid. Ba^2+^ was used as charge carrier, replacing the Ca^2+^ ions, to avoid Ca^2+^‐dependent inactivation and the activation of Ca^2+^‐activated Cl^−^ channels (Cl^−^_Ca_), known to be endogenously expressed in oocyte membrane 45. We have recently demonstrated in identical experimental system that blocking the Cl^−^_Ca_ current does not interfere with the HP effect on VDCCs 30. Both Ca_V_2.1 and Ca_V_2.2 also have higher conductance to Ba^2+^ 46, allowing measurement of minute currents that otherwise would have been unnoticed. The solution, saturated with air at atmospheric pressure, was introduced into the chamber by the use of a high‐pressure pump (Minipump; LDC Analytical Inc., Riviera Beach, FL, USA) at room temperature (24–25°C), at a rate of 1.5–2 ml/min. Temperature was constantly monitored throughout the experiments by the use of a thermistor submerged in the solution in the vicinity of the oocyte groove. Deviation of only ±0.5°C was allowed from the control temperature for later measurements. We have also demonstrated in our recent study 30 that the small reversible adiabatic temperature changes are not responsible for the response of the VDCCs to HP. In addition, we have proved that the voltage and currents measurements in our setup are stable along the relatively long duration of compression and decompression. Typical recorded traces are shown in Figure 1. Voltage traces are not ‘command voltages’ but rather the actual recording of the oocyte transmembrane potential. Holding potential was −80 mV (see example in Fig. 1A). The duration of each depolarizing step was 500 msec., which was preconditioned by a 100‐msec. hyperpolarizing step to −90 mV to release the VDCC from partial inactivation. The latter was also used to calculate and monitor the oocytes' instantaneous input resistance for measuring and subtracting the leak currents, which were accounted for at each recorded trace separately, thus unmasking the net VDCC current.

Every series of depolarizing pulses was used to construct an I‐V curve and repeated at least three times to verify stability of the currents, as was previously described (fig. 1 in 30). Recorded traces with voltage fluctuation greater than 2 mV during depolarization were disregarded. We studied HP effects on I‐V curve, maximal currents, activation and inactivation functions, channel kinetics such as time to peak (TTP) and time constants (τ), and voltage dependency. Maximal currents were measured at the minimal point of the current curve. Inactivation (I/Imax) was measured towards the end of the depolarizing step in comparison to the measured maximal current (as above). A fit was calculated for each decaying section of the current in every recorded trace according to a biexponential equation 47 defining two time constants for decay:

$$\text{Fit}=-A1\exp\left(-t/\tau_{\mathrm{Decay}\;\;\mathrm{Fast}}\right)-A2\exp\left(-t/\tau_{\mathrm{Decay}\;\;\mathrm{Slow}}\right)+C$$

For the rising phase and the tail currents, a single exponential fit was performed. All fits were calculated between the curves' normalized values of 0.1 and 0.9.

Activation volume (Δ*V* ^‡^) was calculated for time constants of channel activation, inactivation and deactivation under normobaric and hyperbaric conditions, following the known equation 48:

$$\Delta V^{‡}=RT{\left(\partial \ln\tau /\partial P\right)}_{T}$$

### Helium compression

After control measurement taken at 0.1 MPa, compression steps to 0.5, 2.5 and 5.0 MPa were performed by compressed helium. Compression was done manually at a rate of approximately 0.25–0.5 MPa/min. and never exceeded 1.0 MPa/min. Helium was used instead of air due to its inert quality and the need to avoid known nitrogen narcosis and oxygen toxicity–related effects 49. Principally, compression with helium does not change the other gases (primarily oxygen and nitrogen) partial pressure. Here, the chamber gaseous content was flushed with helium during compression due to the need to drain the excess of physiological solution, and thus the oxygen and nitrogen partial pressure was reduced over time. However, the oocytes were continuously perfused with fresh solution equilibrated with air at 0.1 MPa, and thus the oocytes were exposed to normal partial pressure of oxygen and nitrogen. All pressure units are absolute.

### Statistical analysis

**Figure 2:**
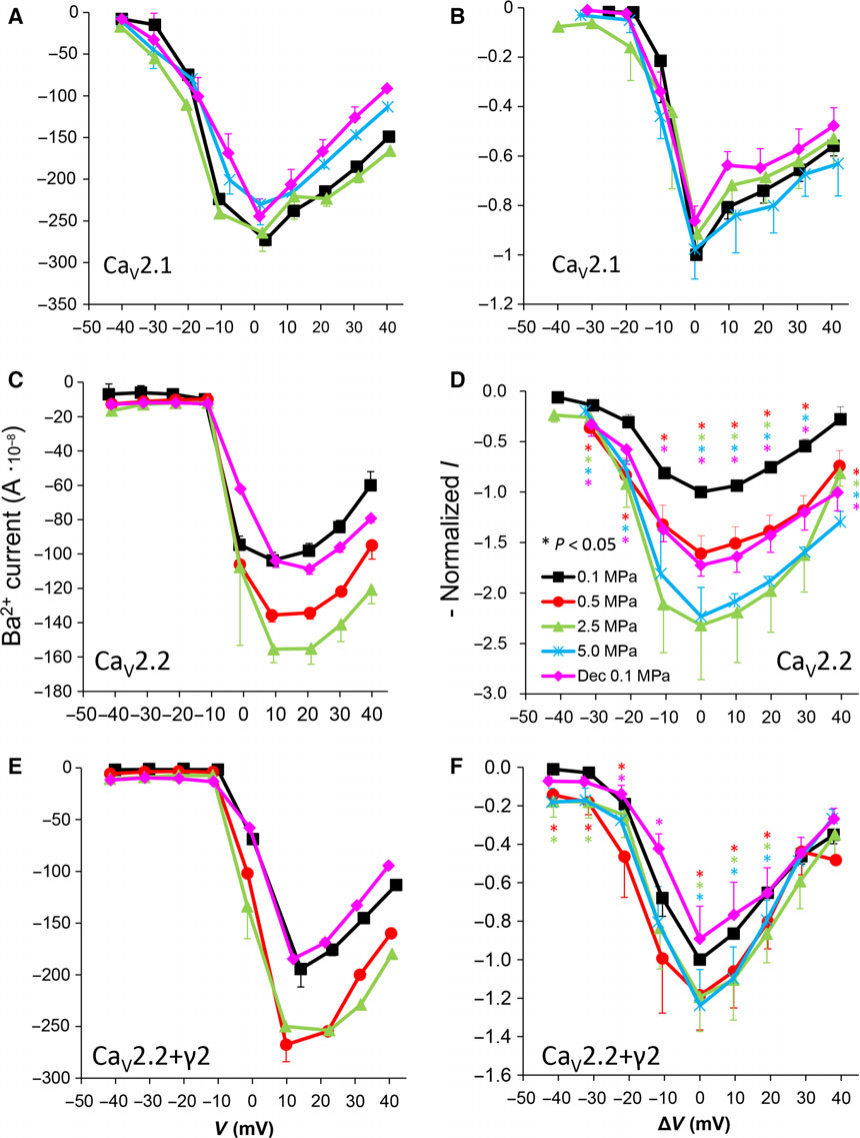
I‐V curves of maximal currents. (**A** and **B**) Ca_V_2.1, (**C** and **D**) Ca_V_2.2, (**E** and **F**) Ca_V_2.2_+γ2_ channels. (**A**,** C** and **E**) I‐V curve of a single oocyte. (**B**,** D** and **F**) Pooled data from 7–9 (**B**), 9–12 (**D**) and 7–10 (**F**) oocytes exposed to 0.5–5.0 MPa pressure (colour indicated), normalized to maximal current at 0.1 MPa, holding potential is adjusted [Δ*V* (mV)] so that 0 indicates the potential at which maximal current is obtained (V_Imax_). Statistical significance for each point on the curve is indicated by corresponding colour asterisks (*P* < 0.05). Dec indicates decompression.

**Figure 3:**
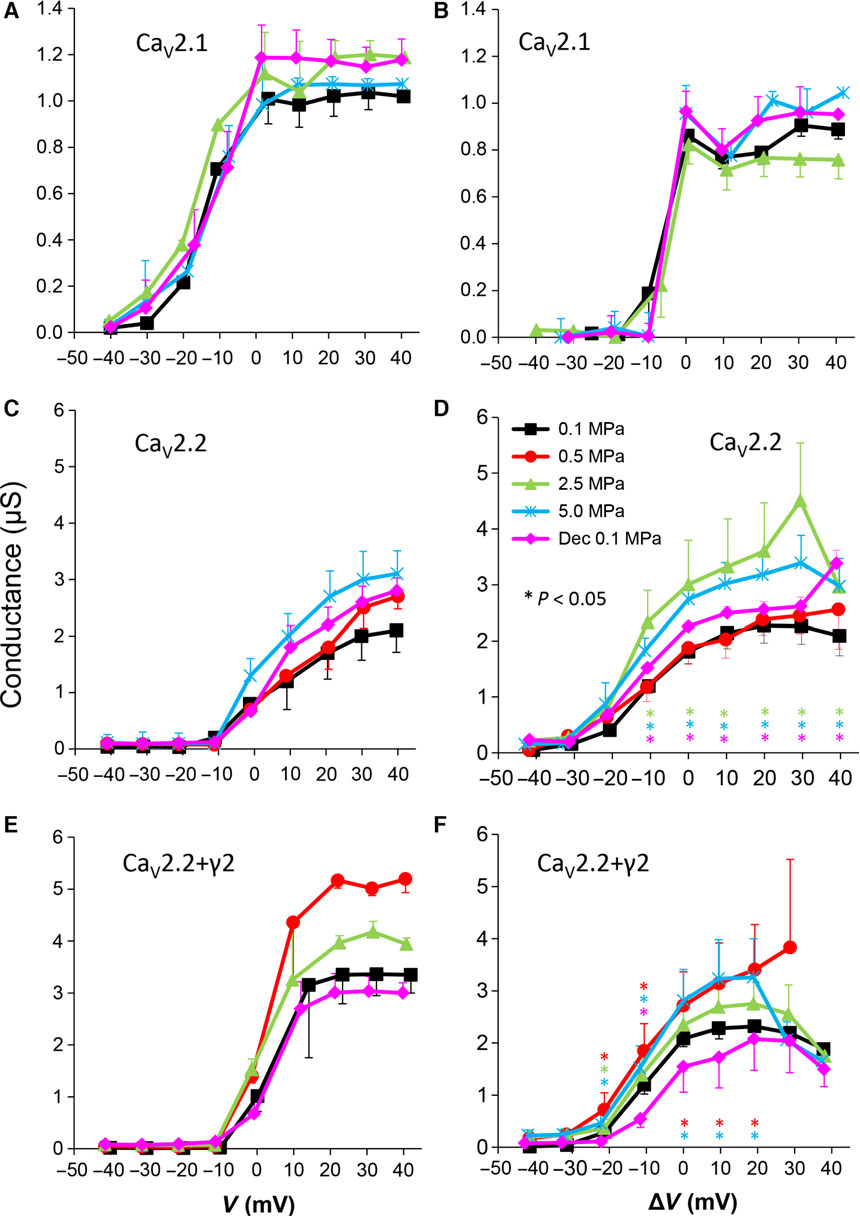
Channels' conductance at various pressures. (**A** and **B**) Ca_V_2.1, (**C** and **D**) Ca_V_2.2 and (**E** and **F**) Ca_V_2.2_+γ2_ channels. (**A**,** C** and **E**) Conductance measured in a single oocyte. (**B**,** D** and **F**) Pooled data of the channels, *n* as stated in Figure 2. Statistical significance for each point on the curve is indicated by corresponding colour asterisks. Holding potential [Δ*V* (mV)] is expressed as in Figure 2. Dec indicates decompression.

**Figure 4:**
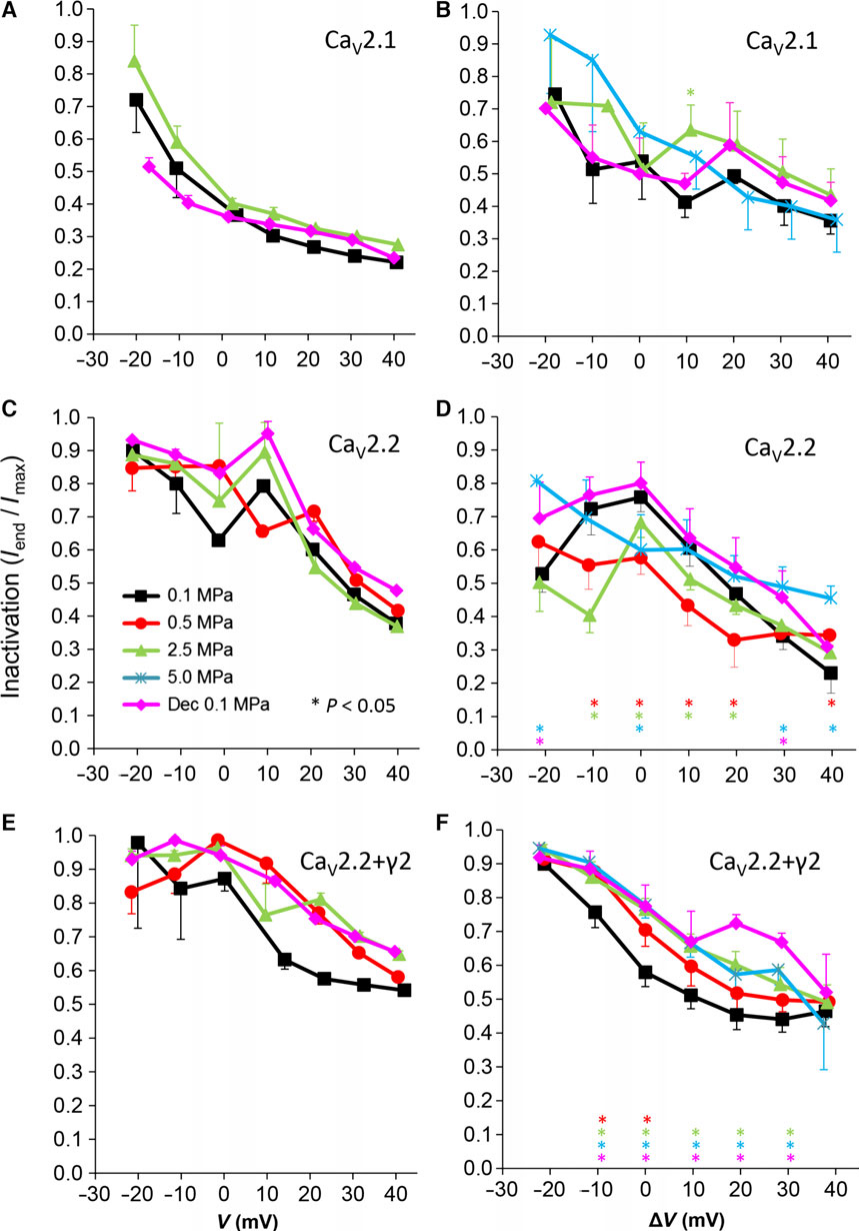
Voltage‐ and time‐dependent current inactivation (I_end_/I_max_) at various pressures. (**A** and **B**) Ca_V_2.1, (**C** and **D**) Ca_V_2.2 and (**E** and **F**) Ca_V_2.2_+γ2_ channels. (**A**,** C** and **E**) Inactivation measured in a single oocyte. (**B**,** D** and **F**) Pooled data of the channels, *n* as stated in Figure 2. Pressures are colour indicated. Statistical significance for each point on the curve is indicated by corresponding colour asterisks. Holding potential [Δ*V* (mV)] is expressed as in Figure 2. Dec indicates decompression.

**Figure 5:**
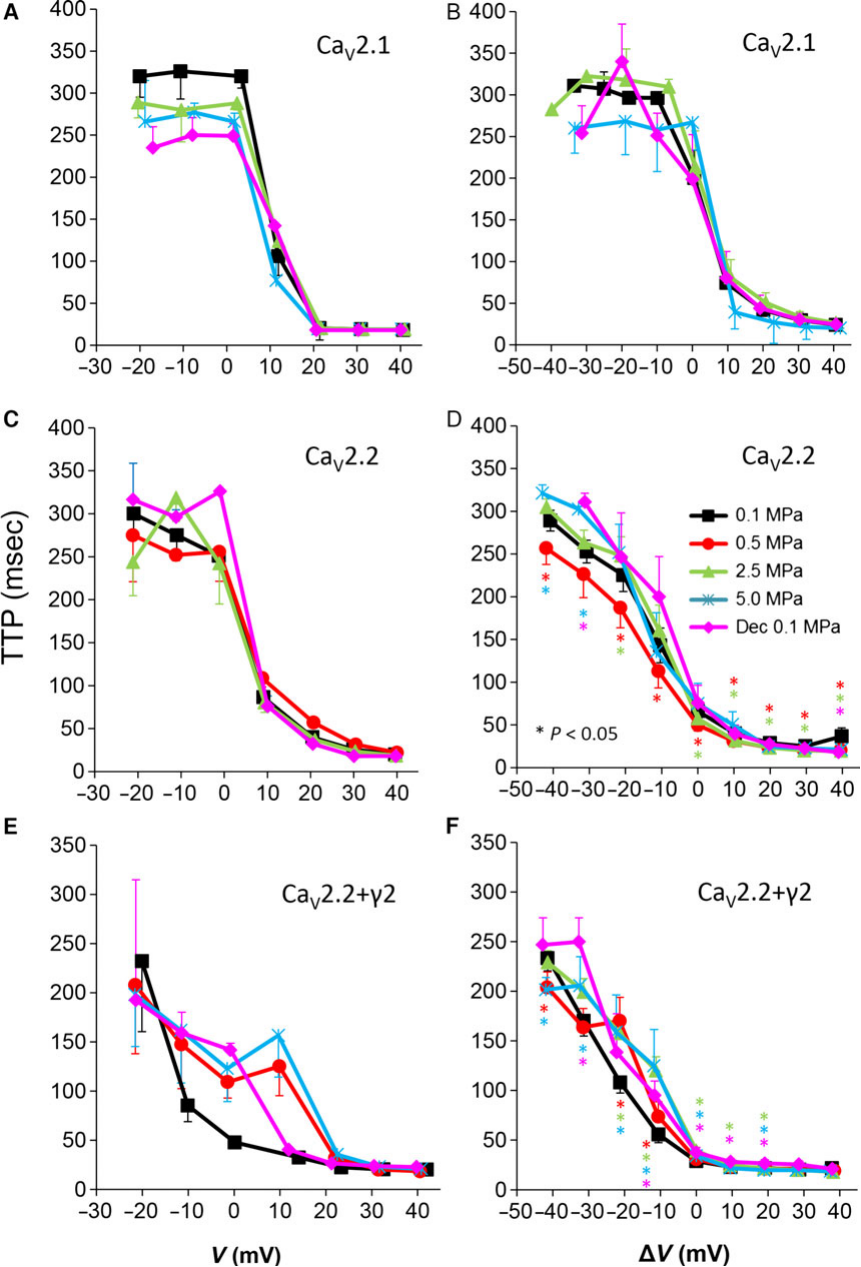
Time to current peak (TTP) from stimulus onset at various pressures. (**A** and **B**) Ca_V_2.1, (**C** and **D**) Ca_V_2.2 and (**E** and **F**) Ca_V_2.2_+γ2_ channels. (**A**,** C** and **E**) TTP measured in a single oocyte. (**B**,** D** and **F**) Pooled data of the channels, *n* as stated in Figure 2. Pressures are colour indicated. Statistical significance for each point on the curve is indicated by corresponding colour asterisks. Holding potential [Δ*V* (mV)] is expressed as in Figure 2. Dec indicates decompression.

**Figure 6:**
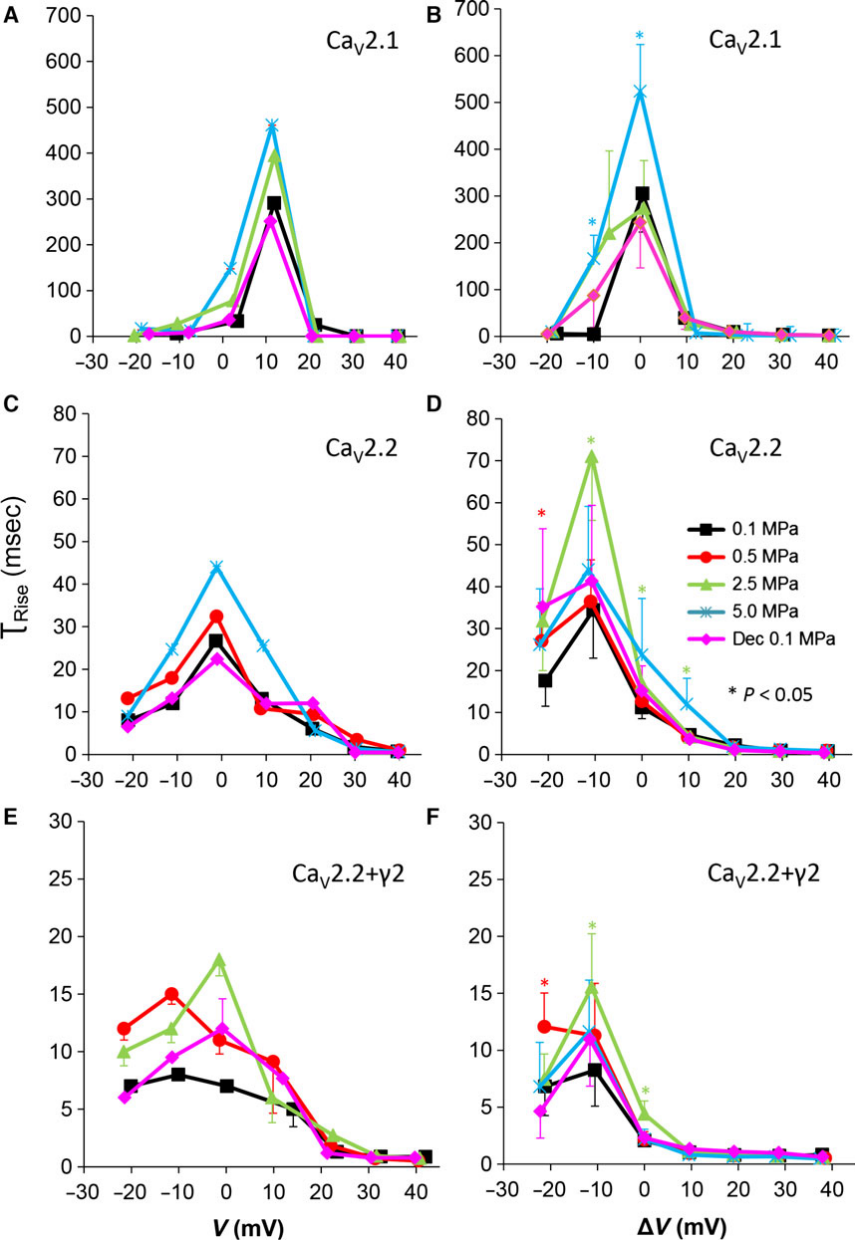
Time constant of current activation (τ_Rise_) at various pressures. (**A** and **B**) Ca_V_2.1, (**C** and **D**) Ca_V_2.2 and (**E** and **F**) Ca_V_2.2_+γ2_ channels. (**A**,** C** and **E**) τ_Rise_ measured in a single oocyte. (**B**,** D** and **F**) Pooled data of τ_Rise_, *n* as stated in Figure 2. Pressures are colour indicated. Statistical significance for each point on the curve is indicated by corresponding colour asterisks. Holding potential is expressed [Δ*V* (mV)] as in Figure 2. Dec indicates decompression.

**Figure 7:**
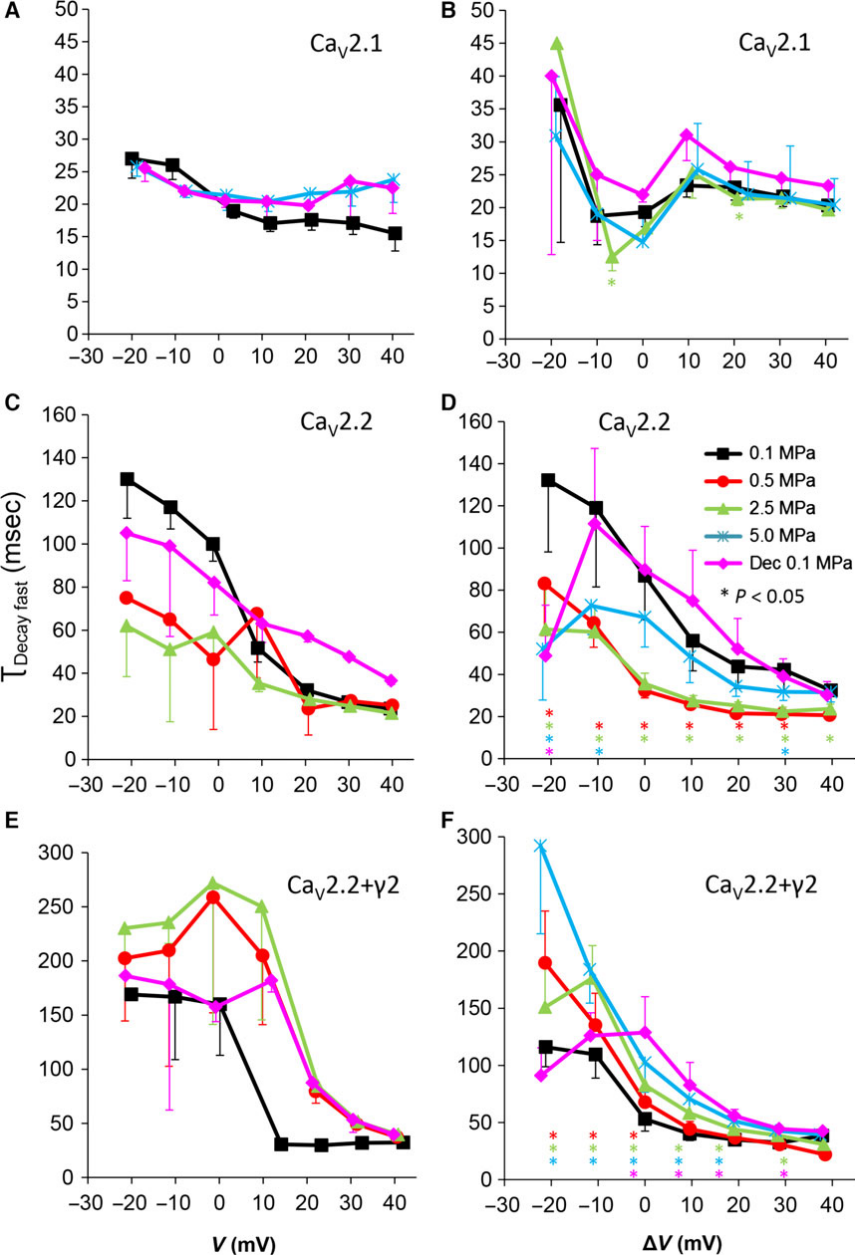
Fast time constant of voltage‐ and time‐dependent current inactivation (τ_Decay Fast_). (**A** and **B**) Ca_V_2.1, (**C** and **D**) Ca_V_2.2, (**E** and **F**) Ca_V_2.2_+γ2_ channels. (**A**,** C** and **E**) τ_Decay Fast_ measured in a single oocyte. (**B**,** D** and **F**) Pooled data of the channels, *n* as stated in Figure 2. Pressures are colour indicated. Statistical significance for each point on the curve is indicated by corresponding colour asterisks. Holding potential [Δ*V* (mV)] is expressed as in Figure 2. Dec indicates decompression.

**Figure 8:**
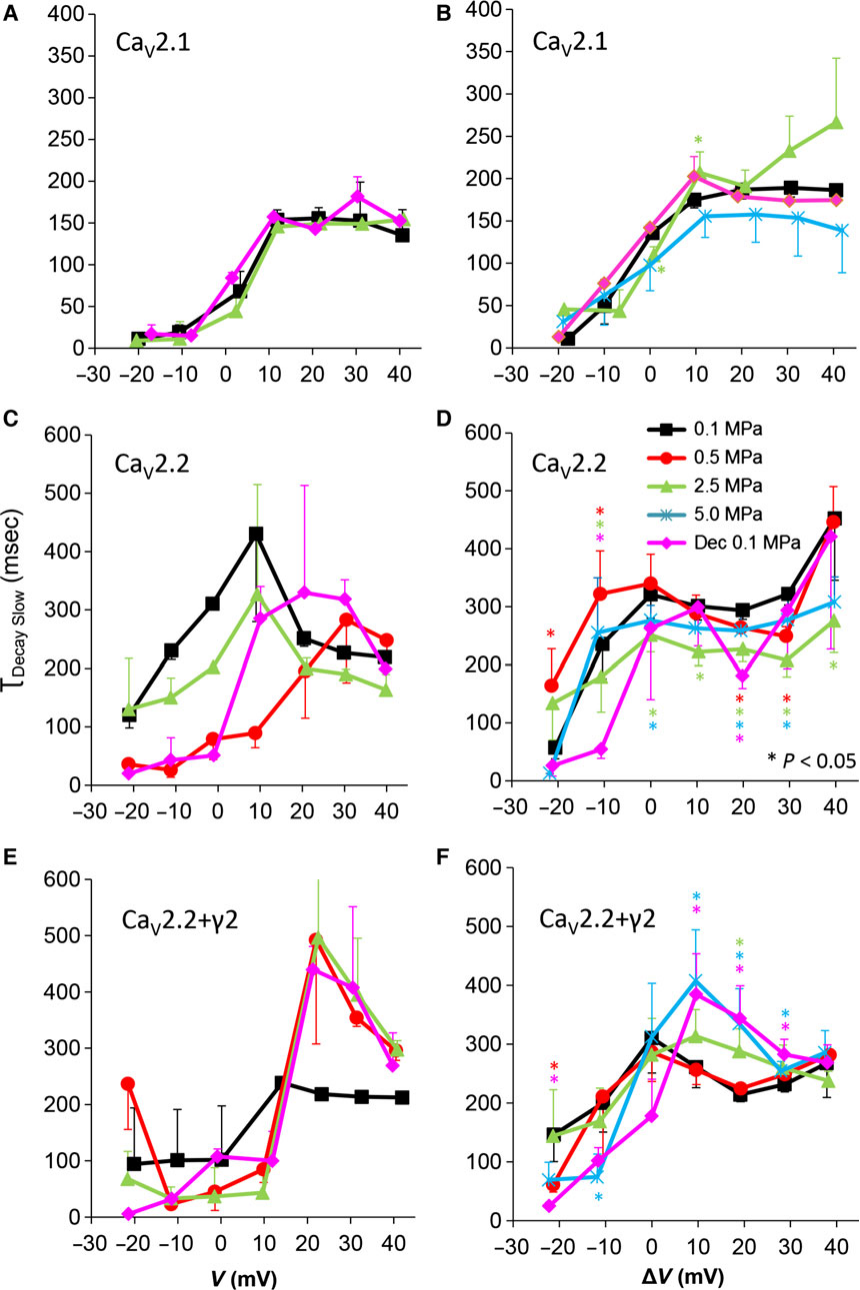
Slow time constant of voltage‐ and time‐dependent current inactivation (τ_Decay Slow_). (**A** and **B**) Ca_V_2.1, (**C** and **D**) Ca_V_2.2 and (**E** and **F**) Ca_V_2.2_+γ2_ channels. (**A**,** C** and **E**) τ_Decay Slow_ measured in a single oocyte. (**B**,** D** and **F**) Pooled data of the channels, *n* as stated in Figure 2. Pressures are colour indicated. Statistical significance for each point on the curve is indicated by corresponding colour asterisks. Holding potential [Δ*V* (mV)] is expressed as in Figure 2. Dec indicates decompression.

**Figure 9:**
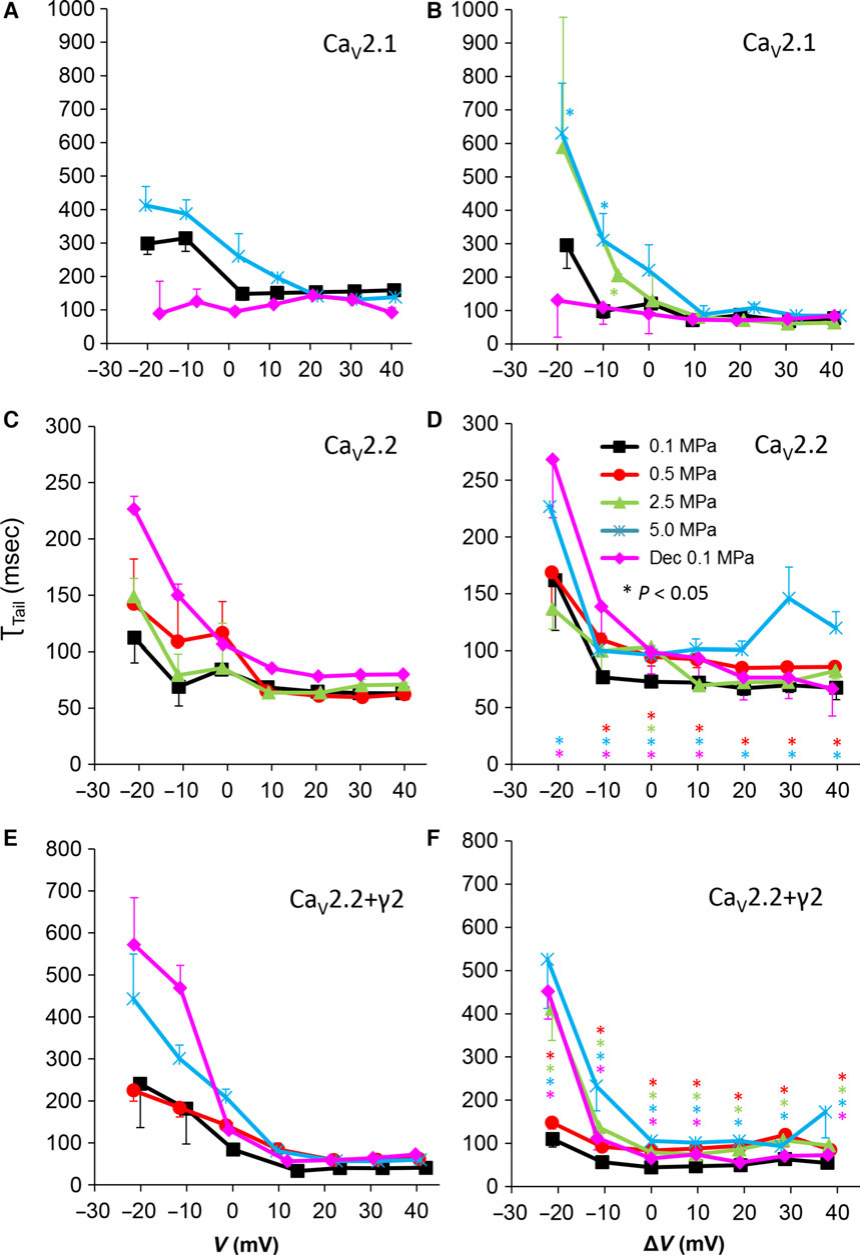
Tail current time constant (τ_Tail_) at various pressures. (**A** and **B**) Ca_V_2.1, (**C** and **D**) Ca_V_2.2 and (**E** and **F**) Ca_V_2.2_+γ2_ channels. (**A**,** C** and **E**) τ_Tail_ measured in a single oocyte. (**B**,** D** and **F**) Pooled data of τ_Tail_, *n* as stated in Figure 2. Pressures are colour indicated. Statistical significance for each point on the curve is indicated by corresponding colour asterisks. Holding potential [Δ*V* (mV)] is expressed as in Figure 2. Dec indicates decompression.

The full set of parameters was calculated off‐line for each recorded trace separately, considering the instantaneous input resistance and leak currents where appropriate, using a dedicated self‐designed Matlab software program. The data were exported to Microsoft Excel software (Microsoft Corp., Redmond, WA, USA). Repetitive measurements of I‐V curves, verifying stability of the measured currents, were averaged and used as a single value for each depolarization step, which in turn was used for averaging with results from other oocytes. The same was done for all other parameters. Each oocyte was used as its own control, and thus values were normalized to 0.1 MPa when needed. When data from more than one oocyte were pooled, binning was performed relative to the voltage generating the maximal current in the I‐V curve (V_Imax_); hence, in figures representing these data (Figs 2, 3, 4, 5, 6, 7, 8, 9B, D and F), the *X*‐axis title is Δ*V*. The actual X values in all figures were determined by averaging the actual recorded voltages during depolarizing steps. Hence, minor shifts of 1–2 mV from the values indicated in the *X*‐axis may occur. Paired sample *t*‐test was used to analyse the significance of the results: each pooled value was compared with its pertinent pooled value at 0.1 MPa for the same Δ*V*. Significant difference (*P* < 0.05) is represented by asterisks in figures.

## Results

### Unaffected current in Ca_V_2.1

As expected, the amplitude of Ba^2+^ currents in Ca_V_2.1 was not affected in oocytes exposed to HP (2.5–5.0 MPa, see example in Fig. 2A). Comparing the normalized maximal currents (negative peak in I‐V curve) at V_Imax_ shows that compression to 2.5 and 5.0 MPa did not significantly change the maximal currents (−8 ± 10% and −2 ± 3%, respectively, *P* > 0.4, *n* = 7–9, Fig. 2B). Decompression to 0.1 MPa also did not significantly affect the maximal current; it remained slightly depressed by −14 ± 6% (*P* > 0.3, *n* = 6). Neither the threshold voltage nor V_Imax_ were affected by HP.

### Augmented current in Ca_V_2.2

Surprisingly, Ba^+^ currents in Ca_V_2.2 were significantly increased at HP (0.5–5.0 MPa) in a dose‐dependent manner (see example in Fig. 2C), in contrast to the expectations based on previous studies (see Introduction). Compression to 2.5 and 5.0 MPa caused a similar augmentation of the maximal currents at V_Imax_ by 132 ± 54% and 123 ± 8% of control values, respectively (Fig. 2D, average ± SEM, *P* < 0.01, *n* = 7–9); therefore, lower HP steps to 1.1 MPa and 0.5 MPa were performed in subsequent experiments in order to reveal the threshold for HP effect. However, the maximal current at 1.1 MPa was augmented in a similar manner by 122 ± 56% (*P* < 0.01, *n* = 3, data not shown), and only a lower HP perturbations to 0.5 MPa had a weaker effect on the maximal currents at V_Imax_: a 61 ± 16% augmentation (*P* < 0.05, *n* = 12). Neither the threshold voltage nor the V_Imax_ were affected by HP. Decompression to 0.1 MPa only partially recovered the current, which remained augmented by 73 ± 15% (*P* < 0.05, *n* = 7).

### Ca_V_2.2 expressed including the γ_2_ subunit

The functionality of a recombinant channel is vastly dependent on the subunits constructing it, their type and isoforms, *etc*. The unexpected HP‐induced current augmentation in the Ca_V_2.2 led us to speculate whether this recombinant channel is affected differently by HP than the native one. In order to elucidate this issue, we have repeated the experiments following expression of the Ca_V_2.2 including the one subunit that was excluded thus far as it is not essential for the channels' functionality, the γ_2_. Although identifying it as a classic subunit of this channel is still debatable, its role in modulating it is not 38, 50.

Indeed, the Ca_V_2.2 expressed including the γ_2_ subunit (Ca_V_2.2_+γ2_) has reacted differentially to HP perturbation. For example, the augmentation of currents witnessed in the Ca_V_2.2 was substantially subsided (Fig. 2E and F).

### Channels' conductance

The cumulative conductance for the population of the channels (‘input conductance’ of the oocyte) calculated relatively to the membrane potential shows similar results to the general findings in the I‐V curves (see examples in Fig. 3A, C and E). High HP (2.5–5.0 MPa) increased the conductance in the Ca_V_2.2 and Ca_V_2.2_+γ2_ channels (Fig. 3D and F) but did not have a consistent effect in the Ca_V_2.1 channel (Fig. 3B). On average, the change from threshold to maximal normalized response occurred within a 50‐mV depolarization range for the Ca_V_2.2 and Ca_V_2.2_+γ2_ channels and only 30 mV for the Ca_V_2.1 channel.

### Currents inactivation

We have previously demonstrated that in the absence of Ca^2+^ ions in the solution, the Ca^2+^‐dependent inactivation of these VDCC is eliminated 30, leaving only the voltage‐ and time‐dependent inactivation that can be evaluated as the ratio between the remaining current at the end of the depolarizing voltage step and its maximal value (I_end_/I_max_; see examples in Fig. 4A, C and E). All channels demonstrated a greater inactivation at strong depolarizations, as expected for these VDCCs. For the Ca_V_2.1 channel, HP did not have a consistent or significant effect on inactivation (Fig. 4B). For the Ca_V_2.2 channel, inactivation tended to be stronger when large currents were evoked (Δ*V* −10 to 20 mV) at HP, but was weakened by it around threshold voltage or towards the reversal potential (*e.g*. Δ*V* −20, 40 mV, respectively). Decompression relieved that effect (Fig. 4D). For the Ca_V_2.2_+γ2_ channel, inactivation was weakened at HP of 2.5–5.0 MPa at the whole voltage range of the channel activity, but only at a narrower voltage range (Δ*V* −10 to V_Imax_) at 0.5 MPa (Fig. 4F). Decompression did not recover inactivation to control values.

### Currents kinetics: time to peak

We have recently demonstrated that HP can affect the kinetics of VDCC current (Aviner *et al*.) 30. If the VDCC kinetic parameters such as the rates of activation, inactivation and deactivation of the current are affected by HP, that may change the maximal current and the total ionic flux through the channel. We have, therefore, measured the time passing from the stimulating depolarizing step to the development of I_max_ (TTP). Examples can be seen in Figure 5A, C and E. Time to peak was not altered by HP in the Ca_V_2.1 channel, excluding a tendency for an increase at V_Imax_ at 5.0 MPa, nor was it changed by decompression (Fig. 5B). It can be seen that a barely threshold depolarization led to a longer TTP value due to the indecisive recruitment of the channels population. For the Ca_V_2.2 channel, the HP effect on TTP was complex. At 0.5 MPa, TTP was decreased; at 2.5 MPa, it was decreased for V_Imax_ and up to Δ*V* 20 mV, but increased below V_Imax_; and at 5.0 MPa, it was slightly increased below V_Imax_ range (Fig. 5D). Decompression recovered TTP to control values. For the Ca_V_2.2_+γ2_ channel, TTP was elongated up to Δ*V* 20 mV, more clearly at high HP (2.5, 5.0 MPa).

### Currents kinetics: τ_Rise_

The time constant of the rising phase of the current, τ_Rise_, is another useful parameter to evaluate the activation of the current (Fig. 6A, C and E). Hyperbaric pressure of 5.0 MPa elongated τ_Rise_ of Ca_V_2.1 at a narrow depolarization range (Δ*V* −10 to V_Imax_), whereas at 2.5 MPa, it showed no significant change (Fig. 6B). The maximal increase in τ_Rise_ of Ca_V_2.2 was at 2.5 MPa at a wider depolarization range (Δ*V* −10 to 10 mV), whereas a smaller change was observed at 5.0 MPa (Fig. 6D). Hyperbaric pressure had almost no statistically significant effect on τ_Rise_ in the Ca_V_2.2_+γ2_ channel. Decompression recovered τ_Rise_ back to control levels in all channels.

### Currents kinetics: fast τ_Decay_

A change in the inactivation value (I_end_/I_max_) could originate from an effect on the channels' rate of decay, as I_end_ is measured at the end of the depolarizing step and not under steady‐state conditions. The decay of VDCCs current is known to have two time constants, fast and slow, which are commonly attributed to voltage and Ca^2+^ inactivation, respectively. However, even with Ba^2+^ as the charge carrier, the decaying current could not be fitted satisfactorily using a single exponent.

For the Ca_V_2.1, as expected by the lack of consistent change in inactivation, HP did not cause a clear change in the fast τ_Decay_ (τ_Decay fast;_ Fig. 7A and B). For the Ca_V_2.2, a considerable shortening of τ_Decay fast_ at HP was observed throughout the activity range of the channel even at 0.5 MPa (Fig. 7C and D), while decompression generally relieved this effect. The HP effect was reversed in the Ca_V_2.2_+γ2_ channel, where τ_Decay fast_ was elongated (Fig. 7F).

### Currents kinetics: slow τ_Decay_

The slow τ_Decay_ (τ_Decay Slow_) in all channels was elongated by stronger depolarizations at 0.1 MPa (Fig. 8A, C and E), similarly to previous findings in VDCCs 30. For the Ca_V_2.1 channel, the τ_Decay Slow_ was almost entirely not affected by HP, as may be predicted by inconsistent effect on its inactivation (Fig. 8A and B). For the Ca_V_2.2 channel, τ_Decay Slow_ was shortened by high HP (2.5–5.0 MPa) at suprathreshold depolarization (Δ*V* −10 mV and above), but compression to lower HP of 0.5 MPa led to a mixed effect: generally elongating τ_Decay Slow_ below V_Imax_ and shortening it above V_Imax_. Decompression eliminated this effect almost entirely (Fig. 8C and D). In the Ca_V_2.2_+γ2_ channel, the effect of high HP (2.5–5.0 MPa) was also reversed, elongating τ_Decay Slow_ above V_Imax_, but low HP (0.5 MPa) had no effect (Fig. 8E and F). Decompression only partially relieved the HP effect.

### Currents kinetics: τ_Tail_

The tail current time constant (τ_Tail_), representing the kinetics of the channels' deactivation, was shortened by increasing depolarization in all channels (see example in Fig. 9A, C and E). Hyperbaric pressure elongated τ_Tail_ in the Ca_V_2.2 and Ca_V_2.2_+γ2_ channels almost throughout their activity range (Fig. 9D and F), but only up to V_Imax_ in the Ca_V_2.1 (Fig. 9B). Decompression recovered τ_Tail_ in Ca_V_2.1, but not so much in the Ca_V_2.2s; τ_Tail_ remained elongated at depolarizations below Δ*V* 20 mV in the Ca_V_2.2 channel and below V_Imax_ in the Ca_V_2.2_+γ2_ channel.

### Activation volume (Δ*V* ^‡^)

Δ*V* ^‡^ values were calculated from the change in the rate of processes under hyperbaric conditions compared with control pressure, as described in the Materials and methods. Δ*V* ^‡^ serves as a tool for assessing the sensitivity of a molecule to pressure perturbation, quantifying it in comparable values. Table 1 summarizes Δ*V* ^‡^ values for the tested VDCCs for 2.5 MPa. Generally, the results correspond to both sensitivity and trend of the changes described above.

**Table 1:** Activation volume values (ml/mole) at 2.5 MPa

Δ*V* ^‡^ (ml/mole)	τ_Rise_	τ_Decay Fast_	τ_Decay Slow_	τ_Tail_
Ca_V_2.1	−111	−134	−223	73
Ca_V_2.2	427	−922	−253	358
Ca_V_2.2_+γ2_	779	455	99	595

A summary of HP effects on these channels is given in Table 2. Overall, Ca_V_2.1 was not significantly affected by HP, whereas the Ca_V_2.2s channels were HP sensitive. Although I_max_ and the conductance showed the same trend, interestingly excluding τ_Tail_, all other parameters measured in Ca_V_2.2_+γ2_ demonstrated an altered response to HP compared with Ca_V_2.2: decreased inactivation value, increased τ_Decay_ _Fast_ and τ_Decay Slow_, and unaffected τ_Rise_ (Table 2).

**Table 2:** General qualitative effect of HP on measured channel characteristics

	I_max_	Conductance	Inactivation	TTP	τ_Rise_	τ_Decay Fast_	τ_Decay Slow_	τ_Tail_
Ca_V_2.1	=	=	=	=	=	=	=	↑(=)
Ca_V_2.2	↑	↑	↑(↓)	↓/↑/=	=/↑/(=)	↓	↓	↑
Ca_V_2.2_+γ2_	↑	↑	↓	↑	=	↑	↑	↑

↑, increase; ↓, decrease; =, no change; ( ), stronger depolarization; /, higher HP.

## Discussion

### Current activation

#### Currents' amplitude

As demonstrated in our previous direct 30 and indirect 15, 20 measurements of currents in VDCCs at HP, pressure effect can be selective. In this study, we report that currents through Ca_V_2.2 are increased, whereas currents through the Ca_V_2.1 channel are generally unaffected by HP. Only a partial recovery in the amplitude of the currents in the Ca_V_2.2 was witnessed on return to atmospheric pressure.

The effect of HP found here on the Ca_V_2.1 channel conforms with previous findings 17, whereas the HP effect on the Ca_V_2.2 is in contrast to previous reports that suggested reduction in Ca^2+^ influx through Ca_V_2.2 40, 41. Considering the fact that the channels tested here are recombinant and comprised human and rabbit genetic material (*versus* native intact lobster and guinea pig preparations), the diversity of VDCCs types and their isoform, the unique HPNS threshold for each animal species and the knowledge that even one amino acid alteration can significantly change the whole protein functionality, this contrast is not necessarily surprising. It, in fact, stresses that the interaction between the channels' subunits may have an impact on the way the channel will react to HP perturbation. In our experiments, we used Ba^2+^ and tested only the voltage‐ and time‐dependent inactivation, whereas the previous findings mentioned above were *in situ*, where Ca^2+^ was the ion carrying the current. If a Ca^2+^‐dependent inactivation of the current, known to be stronger than the voltage‐ and time‐dependent one, is increased at HP, the overall effect could be depression of the maximal current, explaining the difference in the HP effect between the present and previous studies.

On the other hand, the increase in the currents' maximal amplitude in Ca_V_2.2 channel at HP in this work is similar to HP effect found recently in Ca_V_1.2 30 and reminiscent of the ‘delayed rectifier’ K^+^ channels (another member of this protein superfamily) in which the non‐inactivating currents were greater at steady state during HP exposure in invertebrates such as squid 51, 52, 53, snail 54 and lobster 55.

Both Ca_V_2.1 and Ca_V_2.2 channels are mainly expressed at the presynaptic nerve terminals 56, 57 and are involved in neurotransmitters release 58. However, Ca_V_2.2 channel is also expressed in dendrites and cell bodies of neurons, *e.g*. in the rat dentate gyrus 59. In such a case, increased channel activity may augment synaptic release and contribute to ‘dendritic boosting’ (increased transfer function between synaptic inputs and somatic spike generation) previously reported by our laboratory 60. Such boosting, that conforms well to HPNS hyperexcitability, was attributed also to the Ca_V_1.2 channel that is prevalent in the dendrites 30. This process is an example of HP influence on neuronal networks that does not act through synaptic transmission. As mentioned above, increased Ca_V_2.2 currents are quite unexpected. However, the current amplitude in the recombinant Ca_V_2.2_+γ2_ channel, considered to better resemble a native one, was much less affected; the average normalized maximal current in V_Imax_ was increased by ~20% at 2.5–5.0 MPa, compared with ~125% increase for the same pressures of the Ca_V_2.2. Thus, we may assume that some native Ca_V_2.2 channel would be depressed by HP, similarly to the Ca_V_3.2 channel (Aviner *et al*.) 30. At present, we can attribute the synaptic pressure‐resistant module (see Introduction) to the Ca_V_2.1 channel activity; however, we cannot safely attribute the pressure‐sensitive module (reduction in synaptic release) to the activity of any recombinant Ca_V_2.2 channel that we have tested so far. Yet, as both channels are mainly expressed at the presynaptic nerve terminals and are involved in neurotransmitters release 58 (see Introduction), it may be postulated that the individual relative sensitivity or durability to HPNS development in humans may rise from different spatial distribution and quantitative expression of these channels in somatosensory and motor nerves.

#### Channels' conductance

Generally, the calculated conductance (input conductance) behaviour relative to the membrane potential at HP reflects the changes shown in the I‐V curves: unaffected in Ca_V_2.1 and increased in Ca_V_2.2s (Fig. 3B, D and F). However, in the Ca_V_2.2, compression to 0.5 MPa did not increase conductance, despite the augmented current measured. Such a phenomenon could be explained either by altered reversal potential or by changed channel kinetics. A change in the reversal potential, if occurs, will be probably also reflected in the measured conductance at higher HP compressions. This did not happen. However, a faster TTP was measured at 0.5 MPa (see below section), suggesting a mechanism through which elevated total ionic flux could develop without an increase in the steady‐state conductance.

Decompression was successful in the Ca_V_2.2_+γ2_, but only partially recovered conductance in Ca_V_2.2, which was still slightly augmented. Should the conductance remain high for long duration after decompression (presently not tested) in the living organism, that may lead to excitotoxicity of neurons due to high cytosolic [Ca^2+^], which could explain the long‐term cognitive deficits found in veteran occupational deep divers 61, 62, 63, 64.

#### Currents' TTP and τ_Rise_

For the Ca_V_2.1, only a tendency for an elongation of TTP and τ_Rise_ at V_Imax_ at 5.0 MPa was witnessed, whereas in the Ca_V_2.2, there was a mixed response to HP: At low HP (0.5 MPa), TTP decreased, while at high HP (2.5–5.0 MPa), it tended to increase (Fig. 5D). Interestingly, in the Ca_V_2.2_+γ2_, both TTP and τ_Rise_ elongate at HP, without recovery after decompression, similarly to the HP effect reported in VDCCs in frog motor nerve (possibly Ca_V_2.2) 15, guinea pig single cerebellar Purkinje cells (probably Ca_V_2.1) 17 and in isolated Ca_V_1.2 expressed in oocytes 30. The velocity of an action potential was also reduced at HP after a transient increase 16.

Increased measured TTP may also indicate a slower inactivation process, which will make the maximal current appear later. Indeed, the τ_Decay Fast_ was also elongated in Ca_V_2.2_+γ2_ at HP (Fig. 7F, see Current inactivation). Overall, greater ionic flux *via* Ca_V_2.2_+γ2_ could be generated per given depolarization at HP, due to increased conductance and maximal currents and deceleration of inactivation kinetics.

### Current inactivation

A stronger inactivation in the Ca_V_2.2 at HP was also supported by shorter τ_Decay Fast_ and τ_Decay Slow_ (Figs 7 and 8). Interestingly, at 5.0 MPa, stronger depolarization (Δ*V* >20 mV) weakened the inactivation, suggesting the HP effect is also dependent on the currents' driving‐force, *i.e*. membrane potential.

Almost no significant effect of HP on inactivation value was measured in the Ca_V_2.1, excluding some changes at 2.5 MPa but with marginal *p* values, also in τ_Decay Slow_ (Fig. 8). This seems to be a non‐linear HP effect, as was previously found in other VDCCs 30.

In the Ca_V_2.2_+γ2_, inactivation was weaker at HP throughout the activity range of the channel, which correlated with elongation of both τ_Decay Fast_ and τ_Decay Slow_ (Fig. 4). This is an opposite finding to the result in the Ca_V_2.2, which may suggest that the γ2 subunit has a role in the inactivation process of the naïve channel and also the sensitivity of the molecular mechanism controlling the voltage‐dependent inactivation to HP. Since the Ca_V_2.2_+γ2_ may represent a more ‘native’ channel, this result also conforms with the slower inactivation at HP that was reported in Na^+^ channel in bovine chromaffin cells 13. The effect of HP on both τ_Decay_s was only at pressures above 0.5 MPa, which is in agreement with the fact that at least 1.0 MPa is needed in order for the HPNS to develop in humans.

Although generally τ_Decay Fast_ and τ_Decay Slow_ were affected similarly by HP for each channel separately (Table 2), both in Ca_V_2.2 and Ca_V_2.2_+γ2_, τ_Decay Slow_ was affected differentially than τ_Decay Fast_ at HP for membrane potentials below V_Imax_ (Δ*V* <0 mV). This further supports the well‐established concept of different mechanisms for the fast and slow inactivation 65, 66, which can also react differently to external treatment 67. It was also demonstrated that the molecular structures responsible for these two types of inactivation are differently located in the VDCC's protein 68 and that the fast inactivation may act similarly to the ‘ball and chain’ mechanism in the K^+^ channel 69, while the slow inactivation seems to be at least partially dependent on the interaction between α_1_ and β subunits 66. As the γ_2_ subunit is known to affect these mechanisms 37, 70 and to interact with Ca_V_β_3_ subunit, both involved in the channels' modulation by Gβγ 38, 71, it is not surprising that the HP effect on inactivation is altered by the presence or absence of γ_2_. The lack of inactivation recovery to control values after decompression in the Ca_V_2.2_+γ2_ suggests that either the conformational changes related to inactivation that γ_2_ is involved in or the interaction site of γ_2_ had been irreversibly altered by HP.

It should be noted that even in the absence of Ca^2+^, still two components of time constants were necessary in order to fit the voltage‐ and time‐dependent inactivating portion of the current. This leads to the notion that the τ_Decay Slow_ described here is also voltage dependent that is usually masked by the relatively faster Ca^2+^‐dependent slow inactivation.

### Currents deactivation

All channels examined responded to HP by elongation of τ_Tail_, whether significantly (Ca_V_2.2 and Ca_V_2.2_+γ2_) or just by a tendency (Ca_V_2.1), implying a slower deactivation at HP (Fig. 9) for these neuronal channels, in oppose to the Ca_V_1.2 30. τ_Tail_ is the only kinetic parameter that was similarly affected in Ca_V_2.2 and Ca_V_2.2_+γ2_ channels, suggesting that the γ_2_ subunit is not involved in the regulation of the deactivation mechanism.

Overall, this fits well with the general pressure effect on the Ca_V_2.2 and Ca_V_2.2_+γ2_ channels witnessed here – an increased flux at HP.

### Activation volume (Δ*V* ^‡^)

Excluding τ_Decay Slow_, all Δ*V* ^‡^ of Ca_V_2.1 are 12–29% of Ca_V_2.2 Δ*V* ^‡^ values. This conforms with the weaker, or even non‐existent, sensitivity of the channel to HP.

All Δ*V* ^‡^ of τ_Tail_ are positive values, indicating a deceleration by HP. This suggests that the deactivation process is similar in the examined channels, although less sensitive in Ca_V_2.1, as mentioned above. Interestingly, τ_Tail_ Δ*V* ^‡^ of Ca_V_1.2 is negative as reported in our recent study 30, suggesting its deactivation mechanism may operate in a different spatial manner.

All Δ*V* ^‡^ of Ca_V_2.2_+γ2_ are positive values, as opposed to the negative τ_Decay_s Δ*V* ^‡^ in Ca_V_2.1, Ca_V_2.2 and even in Ca_V_1.2 and Ca_V_3.2 as also reported in our previous study 30. This indicates that γ_2_ participates in regulation of the inactivation process, a fact that has been revealed by HP exposure.

### Summary

HP hardly affected the behaviour of Ca_V_2.1, but had a major effect in both Ca_V_2.2 and Ca_V_2.2_+γ2_, albeit HP kinetic effect was generally opposite in all aspects but τ_Tail_. These effects may indicate that the conformational changes involved in the channels' activity are facilitated (*e.g*. conductance, τ_Decay fast_ and τ_Decay Slow_ in Ca_V_2.2) or opposed (*e.g*. inactivation and deactivation in the Ca_V_2.2_+γ2_) by an elevated ambient pressure. Indeed, this notion is supported by the calculated activation volumes corresponding to these processes, probably affecting the total ionic flux through the channels at HP.

Some of the effects may indicate a transient or non‐linear nature (*e.g*. TTP and inactivation in Ca_V_2.2, respectively), while other suggested that the HP effect may be reversed by decompression (*e.g*. inactivation, TTP, τ_Rise_, τ_Decay Fast_, τ_Decay Slow_ in the Ca_V_2.2 and τ_Tail_ in Ca_V_2.1, but not TTP and τ_Rise_ in Ca_V_2.2_+γ2_). A qualitative summary of the major HP‐induced findings is given in Table 2. Among these effects, some were dependent on the membrane potential (*e.g*. inactivation in Ca_V_2.2, τ_Tail_ in Ca_V_2.1) or fluctuated at different HP (*e.g*. TTP and τ_Rise_ in Ca_V_2.2).

### General consideration

Although currents in this study were carried by Ba^2+^ ions (and not Ca^2+^, due to the reasons detailed in Materials and methods), we believe that regarding the main aspect of interest in HP influence on VDCC, *i.e*. conductance and amplitude of currents, the HP impact on these parameters reflects the modulation of HP when Ca^2+^ ions are moving through the channels' pore, as was clearly demonstrated in our previous study 30. This, however, does not exclude the possibility that HP may additionally affect Ca^2+^‐dependent mechanisms such as Ca^2+^‐dependent inactivation.

The fact that HP effect was not always consistent in all membrane potentials suggests that one of the pressure targets is the S4 segment in the transmembrane region of α_1_, holding the positively charged amino acids sequence that serve as a voltage sensor, thus affecting any voltage‐dependent mechanisms, *e.g*. activation and inactivation. Hyperbaric pressure interfering with the spatial movement of S4 segment would also cause a change in the gating current. It was indeed demonstrated in the past that a considerable fraction of Δ*V* ^‡^ in activation of Na^+^ channel is associated with gating current 13, 72.

The non‐linear HP effect is reminiscent of a bell‐shaped dose–response curve; a certain pressure causes a maximal effect, while lower or higher pressures weaken it. The TTP and τ_Rise_, that share this behaviour in the Ca_V_2.2, are different parameters for measuring the channels' activation, which is dependent on membrane potential and the successful spatial transformation of the same S4 segment. This transformation requires a strong enough electrical field to cross a certain energetic threshold. It seems that HP influences that threshold in a non‐linear manner (bell‐shaped), suggesting the spatial reorganization to be more complex than one hinge or happening on a single plateau.

Undoubtedly, the changes in both magnitude and kinetics of the response to depolarization at HP would influence these channels' functionality in the living organism, and hence also its motor and cognitive performance. Indeed, the HPNS constellation of sign and symptoms includes changes in EEG, sleep disorders, decrements in locomotor activity, myoclonus and tremors, which may all be expressed as the manifestation of these HP‐induced changes in VDCCs.

We have previously postulated that even a ‘minor’ change made to a section within a subunit 73, 74 or just a single amino acid substitution 75, 76 can significantly alter the VDCC reaction to depolarization, possibly due to a different spatial organization 65, let alone the use of different subunits will have this effect. Naturally, this assumption is supported in the first place by the differential response to HP in the Ca_V_2.1 and Ca_V_2.2, having a different α_1_ subunit comprising the pore and voltage sensor. But further support to this notion is also provided by the differential HP effects in the Ca_V_2.2 and Ca_V_2.2_+γ2_. The saturation of current augmentation at 0.5–5.0 MPa in the Ca_V_2.2_+γ2_ *versus* the dose–response curve of the Ca_V_2.2 may suggest that the γ subunit counteracts the HP effect on the channels' conductance.

We have recently demonstrated HP effects in VDCC 30 and rat‐cultured cortical neurons (unpublished data) already at 0.5 and 0.3 MPa, respectively. Dean & Mulkey (49) have also reported reversible changes in membrane properties in rat medulla solitary complex upon helium compression to as low as 0.3 MPa. Relatively low HP threshold, 0.5 MPa, was also found here in the Ca_V_2.2 and Ca_V_2.2_+γ2_.

Since HP can target either the channel (and its subunits) or any external modulator, and although the general impression from this study is that HP affects the channel itself by changing its spatial organization in the active or non‐active states, further research is needed to determine whether VDCCs' modulators are also affected by HP. Notwithstanding, ion channel configuration may also be affected by the membrane characteristics (*e.g*. fluidity, input resistance, specific capacity), which have been shown to be affected by HP 55, 77, 78, 79, even at low HP as well (<0.4 MPa) 80. Barosensitivity is commonly attributed to be a manifestation of pressure equally exerted in all dimensions, whereas mechanosensitivity is caused by localized shear and strain forces manifested (at HP) by differences in compressibility of adjacent cellular structures 81, 82. Mechanosensitivity of biological processes has been also demonstrated at relatively low HP (<0.2 MPa) 83, as opposed to barosensitivity of the channel, which usually occurs at high HP (>0.5 and up to 10–40 MPa) 13, 17. This notion may provide another explanation for the non‐linear HP effect: low HP affected the channel *via* altered membrane traits and perhaps mechanosensitivity, while high HP affected the channels itself as well.

On the other hand, a direct influence of HP on the channel may be supported by crystallographic work that has shown the presence of a hydrophobic cavity within a protein, the ability of gas molecules to penetrate it and a reduction in its volume at HP 84, 85. Such a cavity has been proposed to have a role in protein flexibility, which in turn is related to functional efficiency 86. Hence, should a VDCC contain such a cavity, changes in its volume or presence or lack of a gas molecule in it could have a crucial HP‐induced influence on the protein functionality. Such a distortion in the spatial organization and/or conformational change of the channel will also undoubtedly interfere with a prompt recovery back to its naïve state and may provide an explanation for the lack of complete recovery of the channel after decompression in general, and specifically within the time frame of our experiments.

Overall, the direct data being accumulated regarding HP‐induced effects in several types of VDCCs thus far strongly suggest that the previous concept of uniform influence of HP on certain types of channels should be abandoned. As demonstrated by our group, pressure may augment or depress currents, accelerate or decelerate kinetics or leave some of the channels' traits unaffected. The actual mechanism(s) underlying this diversity of responses to HP need further elucidation. Yet, we may speculate that the wide spectrum of pressure sensitivity in vertebrates (*e.g*. tolerance to various levels of HP, while others are obligatory high HP dwellers) is, at least in part, the result of evolutionary differential distribution of these VDCCs throughout neuronal networks, along the single neuron, or structural variations of the same channel in different life forms.

## Conclusions

- HP‐selective modulation of various presynaptic VDCCs (in addition to somatic and dendritic channels) probably has an important role in synaptic transmission alteration, which is strongly associated with HPNS.
- HP selectivity depends on the different α_1_ subunit comprising the pore and voltage sensor but can also be mediated by other regulatory subunits of the channel protein.
- Pressure modulation of channels' kinetics and function is dependent on the membrane potential.

## Conflict of interest

The authors confirm that there are no conflicts of interest.
